# Treatment of Priapism Secondary to Drugs for Erectile Dysfunction

**DOI:** 10.1155/2019/6214921

**Published:** 2019-08-22

**Authors:** José Pablo Saffon Cuartas, Carolina Sandoval-Salinas, Juan M. Martínez, Héctor A. Corredor

**Affiliations:** Clinical Research Group, Boston Medical Group, Bogotá 110111, Colombia

## Abstract

Priapism may present as a side effect in patients treated with medications for erectile dysfunction, in which it should be controlled in a timely manner to avoid complications. There is little information regarding the use of local measures for the treatment of this condition. This study was done with the objective to describe the management of priapism secondary to erectile dysfunction drugs in a cohort of men. Records of emergencies and adverse events were reviewed by two researchers to identify patients diagnosed with erectile dysfunction who received oral or intracavernosal drugs for their illness and presented priapism. Sociodemographic data, clinical background, and information on the duration, management, and evolution of the priapism were extracted. Priapism incidence, percentage of improvement by type of treatment subgroups, and frequency of complications were estimated. 698 patients were treated with PDE-5 inhibitors and 2,135 with intracavernosal drugs. Thirty-one patients (1.4%) reported at least one priapism event during treatment, all with intracavernosal drugs. Treatment with local measures was effective for 10 (32.2%) patients, 1 (3.2%) required terbutaline, 19 (61.2%) used intracavernosal etilefrine, and 1 (3.2%) required drainage and flushing of cavernous bodies. After the priapism episode, 3 (9.6%) patients required an increased dose of the drug in order to achieve satisfactory erection. The results suggest that in men treated for priapism secondary to the use of sexual impotence drugs, initial treatment with local measures and etilefrine can achieve detumescence, decreasing the need for invasive procedures or surgery as a first-line therapy alternative. It is necessary to carry out research studies to confirm this hypothesis.

## 1. Introduction

Priapism is defined as an erection for more than four hours after the erotic stimulation ends or in the absence of stimulation. It is classified as ischemic and nonischemic, the former being more frequent [1, 2]. Its etiology varies and can include blood dyscrasia, metabolic or neurological disorders, infections, toxins, and some drugs including those used to treat erectile dysfunction (ED) [1–4]. Priapism is a very rare event for patients who use phosphodiesterase-5 inhibitors (iPDE-5). An incidence of 0 to 35% has been reported for patients receiving intracavernosal drugs, depending on the drug used and how the studies define priapism [5–10].

It is widely known that inadequate and late treatment of priapism has irreversible consequences for the patient, secondary to anatomical and functional changes in the cavernous bodies of the penis, which can range from erectile dysfunction to penile fibrosis [1, 2, 4, 11–14]. Lesions are due to the absence of arterial blood flow, which produces an ischemic process that improves only by reducing the erection. Hypoxic and inflammatory changes are evident 12 hours after onset of erection, observing destruction of the sinusoidal endothelium. The exact point at which irreversible damage occurs is difficult to determine [15]. Zacharakis et al. have reported extensive necrosis of cavernous smooth muscle in patients with over 48 hours of priapism [16]. Therefore, timely treatment of this urological emergency is highly important.

There is very little scientific evidence for the use of local measures to treat priapism [10, 17]. The last European guidelines for male sexual dysfunction recommended intracavernosal sympathomimetic drugs as a first line of treatment for priapism secondary to intracavernosal injections of vasoactive drugs, while local measures such as ice and exercise, although named, are not recommended because of a lack of scientific support [2].

The aim of this study was to describe the experience in the treatment of priapism secondary to pharmacological treatment of erectile dysfunction in a men cohort attended in a specialized health centre in different cities of Colombia.

## 2. Materials and Methods

This was an observational retrospective study. Institutional records were reviewed to identify patients diagnosed with erectile dysfunction who received oral or intracavernosal treatment, between January 1 and December 31, 2017. In order to determine the priapism cases related with the use of medication, two researchers reviewed: (1) records from call centre emergency services, which describe cases of priapism when patients call to request medical care for this event, and (2) records of adverse events reported by physicians, which describe cases treated at the centers or at other institutions and identified at a follow-up visit.

A priapism case related with the use of medication was defined as an erection for more than 4 hours after sexual stimulation ended after taking a phosphodiesterase-5 inhibitor or intracavernosal injection composed of alprostadil, papaverine, atropine, and phentolamine (QuadMix) or alprostadil, papaverine, and phentolamine (Trimix); with or without pain, without a history of sickle cell anemia.

After identifying the cases, the researchers reviewed the clinical register of the patients and, entered information regarding duration, treatment, and evolution of priapism in an Excel® database. They also reviewed the records posterior to the presentation of the priapism episode and until the present, as the follow-up period for verifying sequels.

The data was validated before the analysis by a third investigator. When extreme or incoherent data were found, the clinical history was reviewed to confirm the information. Absolute and relative frequency measurements were calculated for categorical variables, and measures of central tendency and dispersion were estimated for numerical variables, globally and by subgroups of interest according to the duration of priapism. Only the information of the first episode of priapism was included. The incidence of priapism was estimated considering the population at risk as all patients with dysfunction who were treated from January 1 to December 31, 2017, who received pharmacological treatment. Stata 15.1® software was used for statistical analysis.

The study was approved by the institution's research committee and was conducted in accordance with ethical considerations for human research described by the Declaration of Helsinki and by national norms, respecting the rights and confidentiality of the research subjects.

## 3. Results

During the period of interest, 2,833 patients with erectile dysfunction were treated with medication, of which 698 (24.7%) received PDE-5 inhibitors as a first line of treatment. Another 2,135 (75.3%) received intracavernosal drugs as a second-line therapy option. Thirty-one (1.4%) patients with intracavernosal treatment reported at least one priapism event; thirty with QuadMix and one with Trimix. Four patients presented two episodes of priapism and one man presented 3 incidents. None of the patients receiving oral drugs reported an erection for more than 4 hours after sexual stimulus. One patient presented priapism with the first application of the drug on the erection test; in the other cases, patients presented this condition during the treatment.

For the group of men who presented priapism, the average age was 48.6 years (SD 11.7 years). The most frequent comorbidity was high blood pressure (eight patients), nineteen patients consumed alcohol occasionally, and seven were smokers. The median duration of the ED was 4.4 years, and the median IIEF-5 score at the time of diagnosis was 14 points. The dose of the medication varied between 6 and 54 units (Table 1).

**Figure 1:**
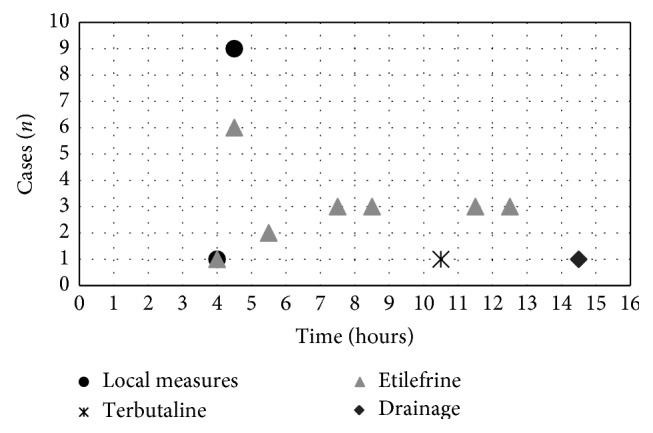
Number of patients by measures applied and duration of priapism.

Regarding to the first priapism event related with the use of medications, the median erection time was 4.5 hours (range 4 to 14.5 hours), 4 patients (12.9%) had priapism for more than 12 hours. The application of local measures (cold water or ice on the penis, and perianal area) or exercising, resulted in detumescence in 10 patients (32.3%), all of whom had an erection lasting less than 5 hours. One patient (3.2%) required additional terbutaline after 6 hours of priapism, which resulted in detumescence 4 hours after taking the drug (this was used due to a lack of access to etilefrine). Nineteen subjects (61.3%) required intracavernosal administration of etilefrine, 68.4% of this took a dose of 10 mg or less to reverse priapism. One patient (3.2%) required drainage and flushing of cavernous bodies after 11 hours of erection, since etilefrine and terbutaline were not available given the remote region where he was located and it is not possible to know if it would have responded to less invasive measures ass etilefrine. No patients underwent surgical intervention (Figure 1).

**Figure 2:**
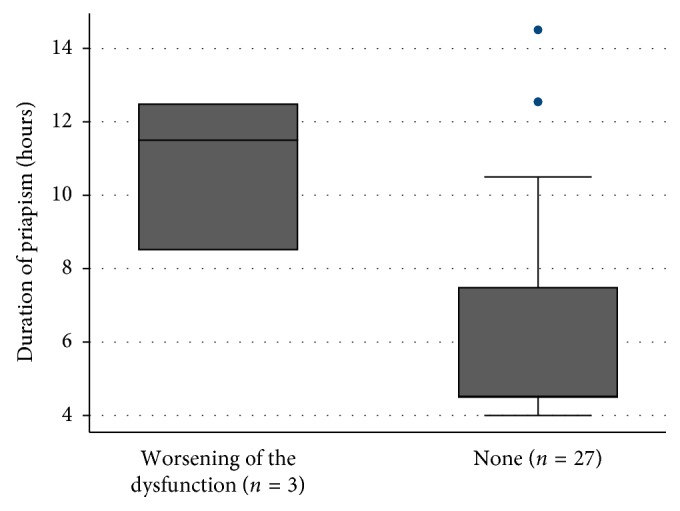
Sequels and duration of priapism.

In the follow-up, the medication dose was increased or the treatment formula was changed in 3 patients (9.7%), which may suggest deterioration in erectile function. Twenty-seven patients (87.1%) did not report any event and continued their treatment with a dose equal to or less than before the event (Figure 2). Follow-up information was not obtained from 1 patient.

## 4. Discussion

Priapism secondary to erectile dysfunction medications is an event of great interest to patients and physicians, especially because of the consequences when not treated early or adequately, such as fibrosis of cavernous bodies and permanent erectile dysfunction [14].

In the present study, 1.4% incidence of priapism posterior to the use of intracavernous medication was found and 0% after the daily use of phosphodiesterase-5 inhibitors, similar to reports by other studies [6–9, 13].

Current clinical guidelines recommend the use of etilefrine as a first line of treatment for patients with iatrogenic priapism [12]. There is very little evidence to support the use of local measures or exercise to treat this type of priapism, and it is limited to case reports and case series [5, 10, 17]; the majority of the studies focus on pharmacological and surgical treatment [18–21]. We began treatment with local measures, which included exposure to cold water or ice and vigorous physical activity. This was effective in 32.3% of the individuals in the study, a finding that is similar to a report by Habous et al., who indicated decreased erection after climbing up and down stairs for 30 minutes in 39.6% of patients who had prolonged erections (3 hours) after intracavernosal administration of QuadMix for a Doppler [10]. It is important to evaluate whether this option to treat priapism during the first few hours of presentation is being undervalued, given that it can benefit some patients, especially those living in rural areas that are far from a health centre, thereby avoiding the risk of invasive interventions and decreasing medical costs. Regarding intracavernosal etilefrine, this was effective in 61.3% of the cases in our study, including patients with priapism lasting 11.5 to 12. 5 hours.

With regard to sequels, 3 patients experienced increased dysfunction. Nevertheless, given the data recorded, it is not possible to infer as to whether those cases were a consequence of the priapism event or the evolution of the illness itself resulting from a lack of check-ups or other factors, such as diabetes or hypertension, which were presented by 2/3 patients. A limitation of this study was that diagnostic images were not taken to confirm vascular integrity, given that no change was detected by the physical exam and the patients did not report curvatures or masses.

The main limitation of this study was the retrospective collection of data, which can possibly generate information bias [22]. Besides events that are not recorded, this affects the analysis of other variables not found in the historical records and that could be related with priapism or its resolution. In addition, although clinical evaluation did not find fibrous plaque or penile deviations after priapism in any of the patients, Doppler ultrasound was not performed to confirm that there were no sequels of this type.

The results presented herein are limited to the description of our experience, and given the design of the study, causal effects of these interventions cannot be confirmed.

## 5. Conclusions

In patients with priapism secondary to the use of sexual impotence drugs, initial treatment in the first hours with local measures or etilefrine can achieve detumescence, decreasing the need for invasive procedures or surgery as a first-line therapy. It is necessary to carry out studies with more patients and appropriate designs to confirm this hypothesis.

## Figures and Tables

**Table 1:** Clinical characteristics of patients with priapism secondary to erectile dysfunction drugs.

	*N*	%
Comorbidities		
Arterial hypertension	8	25.8
Diabetes	5	16.1
Hyperlipidemia	6	19.4
Peyronie's disease	1	3.2

Consumption history		
Alcohol	19	61.3
Tobacco	7	22.6
Recreational drugs	1	3.2

Characterization of erectile dysfunction	Median	Range
Duration of dysfunction (years)	4.4	4–14
IIEF-5 score	14	2–21

## Data Availability

The data used to support the findings of this study are available from the corresponding author upon request.
